# 3-D radar imaging unlocks the untapped behavioral and biomechanical archive of Pleistocene ghost tracks

**DOI:** 10.1038/s41598-019-52996-8

**Published:** 2019-11-11

**Authors:** Thomas M. Urban, Matthew R. Bennett, David Bustos, Sturt W. Manning, Sally C. Reynolds, Matteo Belvedere, Daniel Odess, Vincent L. Santucci

**Affiliations:** 1 Department of Classics and Cornell Tree Ring Laboratory, Cornell University, Ithaca, NY 14853-3201 USA; 2 Institute for Studies in Landscapes and Human Evolution, Bournemouth University, Poole, BH12 5BB UK; 3 National Park Service, White Sands National Monument, P.O. Box 1086, Holloman AFB, NM 88330 USA; 4 National Park Service, Cultural Resources Directorate, Washington, DC 20240 USA; 5 National Park Service, Geologic Resources Division, Washington, DC 20240 USA

**Keywords:** Palaeontology, Palaeoecology

## Abstract

Footprint evidence of human-megafauna interactions remains extremely rare in the archaeological and palaeontological records. Recent work suggests ancient playa environments may hold such evidence, though the prints may not be visible. These so-called “ghost tracks” comprise a rich archive of biomechanical and behavioral data that remains mostly unexplored. Here we present evidence for the successful detection and 3-D imaging of such footprints via ground-penetrating radar (GPR), including co-associated mammoth and human prints. Using GPR we have found that track density and faunal diversity may be much greater than realized by the unaided human eye. Our data further suggests that detectable subsurface consolidation below mammoth tracks correlates with typical plantar pressure patterns from extant elephants. This opens future potential for more sophisticated biomechanical studies on the footprints of other extinct land vertebrates. Our approach allows rapid detection and documentation of footprints while enhancing the data available from these fossil archives.

## Introduction

Trace fossils in the form of footprints (tracks) occur more frequently in the palaeontological and archaeological records than is commonly assumed. Holocene and Plio-Pleistocene examples have been described at an increasing number of sites and are found primarily in unlithified, erodible substrates^1–5^. Footprints provide evidence of an animal’s presence, pedal anatomy, abundance, co-association with other animals and behavioral ecology, and have been used to infer not only body size and mass, but also pedal anatomy and biomechanics^6^. At some locations, especially in the American southwest, these important yet delicate fossils may hold the key to unanswered questions about human behavior during the upper Pleistocene, particularly those related to hunting activity, with footprints offering access to predator-prey interactions outside the more typical “site” locus of a kill or camp^7^. How researchers detect and record fossil footprints is a burgeoning area of method development in contemporary ichnology^8–11^, and crucial to both maximizing the information yielded and also preserving these fragile traces of the past^12^. Former lake beds and playa sites, which occur extensively across the Americas and in parts of Africa, have the potential to hold these latent ichnological archives.

**Figure 1:**
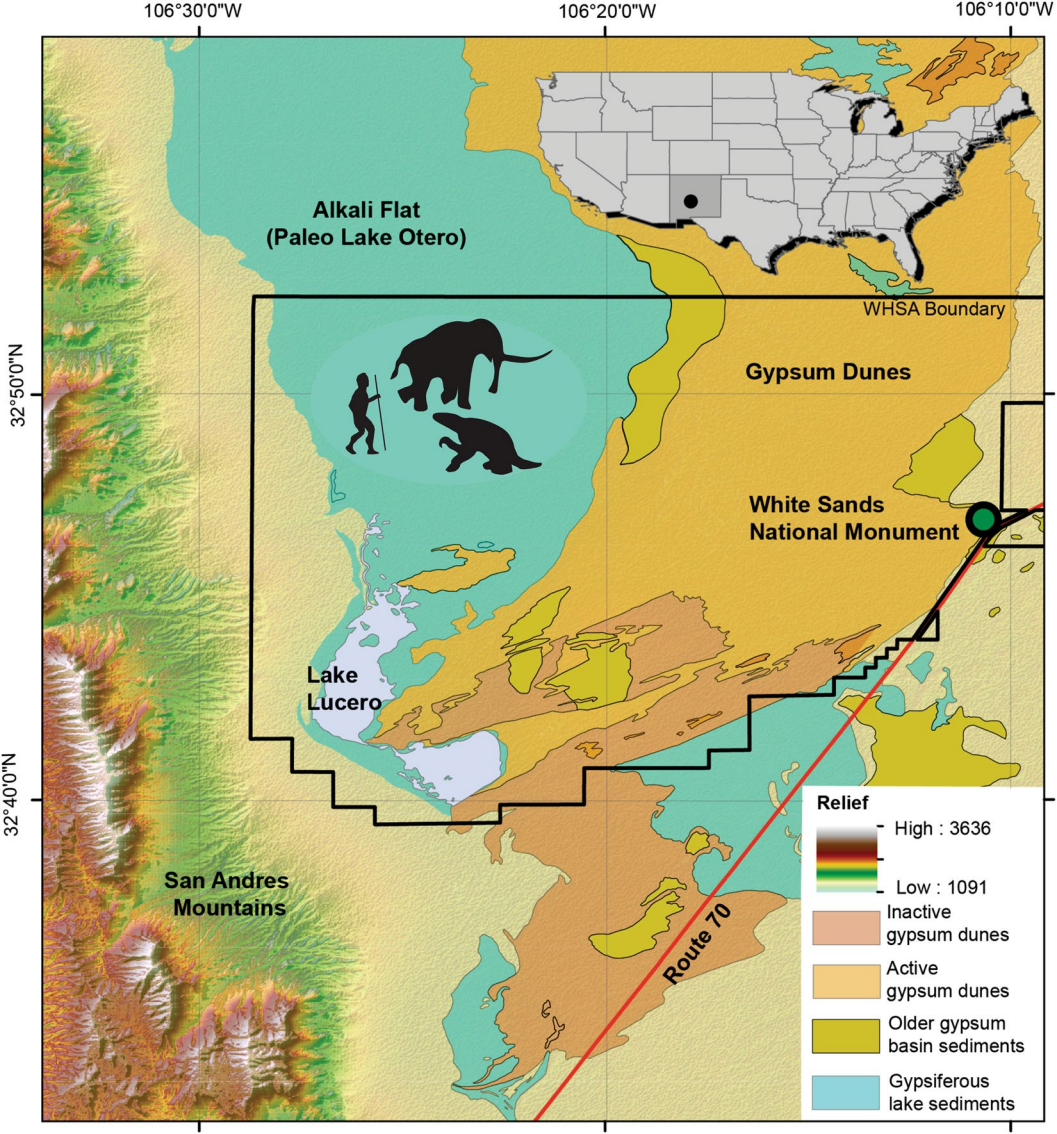
Map showing the White Sands National Monument, Alkali Flat, and the study site. Digital elevation model is from Shuttle Radar Topography Mission 1 arc-second data with the surficial geology taken from U.S. Geographical Survey maps (relief in ft.). Note the precise location of the study site is withheld in accordance with the requirements of the National Parks Service (NPS) compliance with U.S. law. Interested parties may apply to the NPS for the specific site location. Data from https://www.usgs.gov/centers/eros and map made with ArcMap 10.1 (http://desktop.arcgis.com/en/arcmap/).

Here we present findings from White Sands National Monument (WHSA), New Mexico, USA. Our work demonstrates the effectiveness and efficiency of non-destructive GPR for detecting and documenting fossil footprints in soft sediments, including human tracks. Ichnofossils of extinct Rancholabrean fauna occur widely at WHSA and include tracks of Proboscidea (mammoth), Folivora (ground sloth), Carnivora (canid and felid), and Artiodactyla (bovid and camelid), as well as humans. They occur on an extensive gypsum playa (Alkali Flat, Fig. 1), the erosional relict of ancient Lake Otero, dating from the Upper Pleistocene. The sheer number of tracks, tens of thousands extending over large areas, allows animal and human-animal interactions via true ‘paleo-tracking’ to be deduced^7^. This valuable resource however is only intermittently and partially visible at the surface during specific moisture/salt conditions, and when visible may be covered quickly by drifting sand. The occasionally visible tracks are therefore known colloquially as ‘ghost tracks’. Given the scale of the site the resource management challenges are considerable.

The aims of the GPR survey were: (1) to test whether animal tracks, including those of humans, could be detected and usefully resolved with this method, including evaluating the potential of GPR to unpack multiple superimposed track-making events at different horizons; and (2) to explore the 3-D subsurface below larger animal tracks, including how these may be altered by the track-making event. In both cases the geophysical survey was undertaken prior to any excavation or surface preparation with the exception of the removal of loose surface pebbles in order to ensure a smooth traverse for the antenna to improve data quality. We found that GPR (1) allows rapid detection and 3-D recording of multiple species, including humans; and (2) provides non-destructive information on conditions beneath the track of larger fauna, from which we suggest biomechanical inferences may be drawn.

### Site

**Figure 2:**
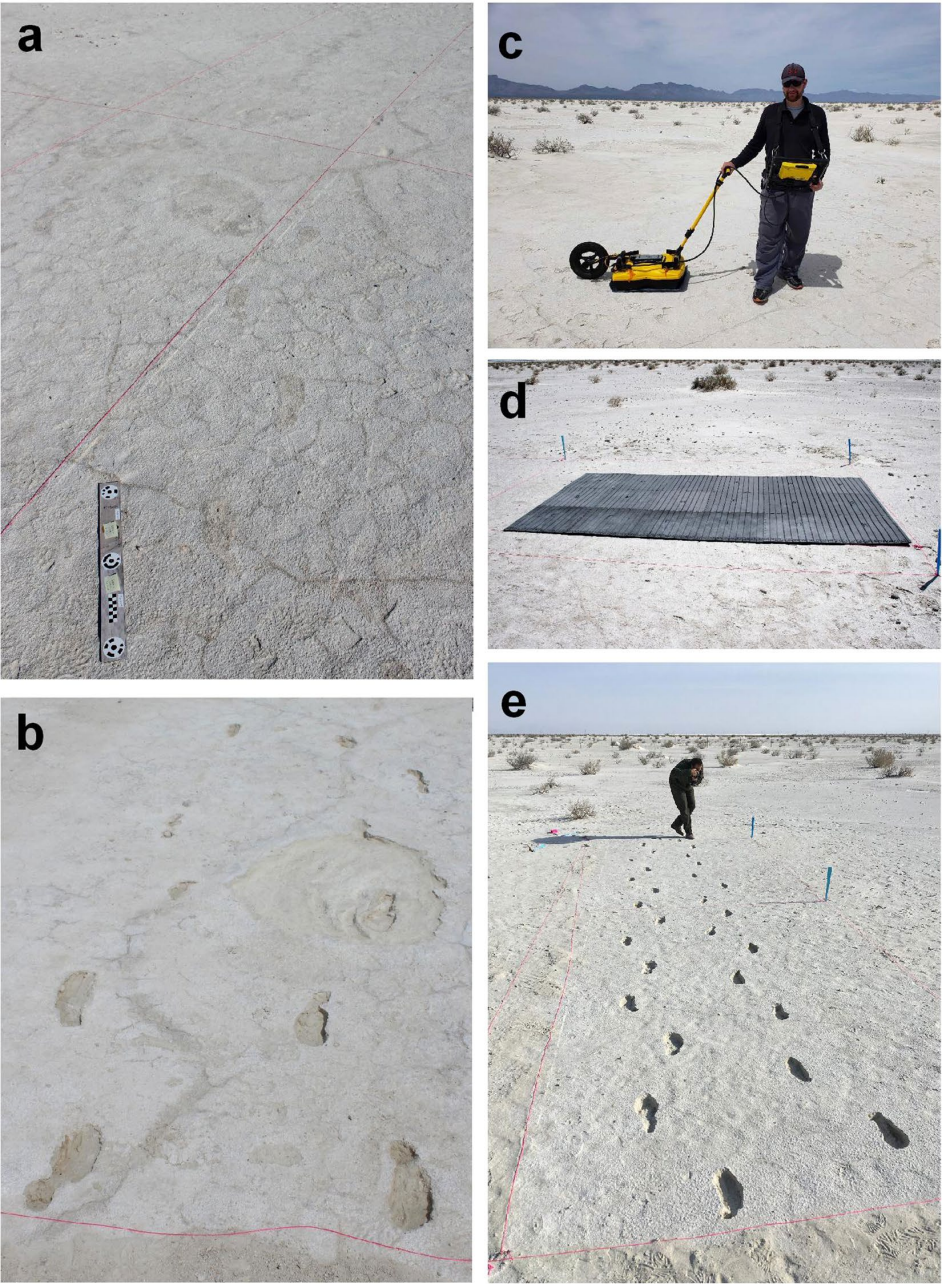
Photographs of the study site at WHSA. (**a**) “ghost tracks”: the surface expression of the tracks is poor as can be seen from the image and they can only be seen under specific moisture and salt conditions. Scale bar 500 mm from target to target. (**b**) Tracks at the study site excavated to reveal both human and mammoth tracks. (**c**) GPR equipment used in this study. (**d**) Gridded foam mats used to protect the surface during the GPR survey following Jacob *et al*.^30^. (**e**) Excavated human tracks at the study site.

**Figure 3:**
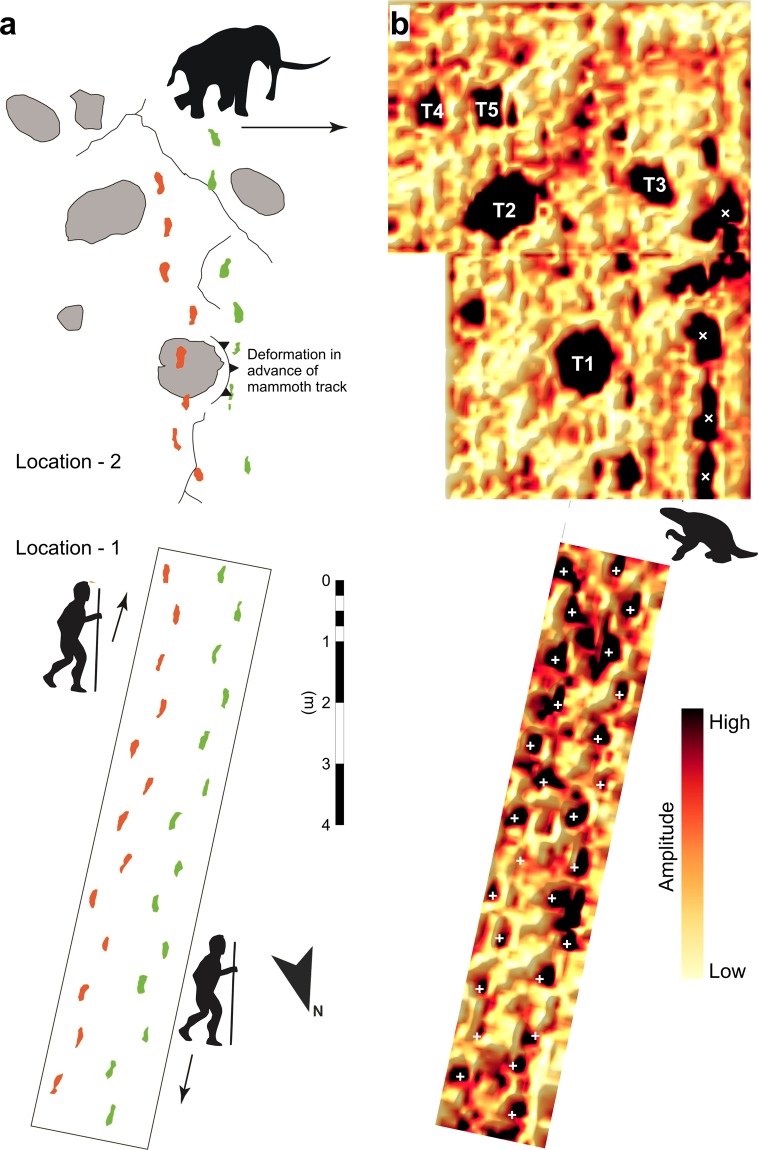
(**a**) The principal tracks and trackways observed at the study site which are split into Location-1 and Location-2 (shown in true spatial relationship). (**b**) GPR amplitude slice (2.0 to 4.0 ns). Human prints that were excavated and used for analysis are indicated with (+) while an unexcavated sloth trackway (identified in subsequent fieldwork) is indicated with (x).

On the eastern side of Alkali Flat there is a double trackway of human prints that extends for over 800 m. This trackway is currently under investigation and appears to represent a single individual walking first north and then returning south after an unknown interval. The two trackways are parallel but off-set by a distance varying between one and two metres. Individual tracks are visible at the surface under appropriate moisture conditions and sections of the trackway have been excavated (Fig. 2). The GPR survey reported here was undertaken along this trackway at a location where a series of proboscidean tracks, presumably *Mammuthus columbi*^13^, cross in a westerly direction perpendicular to the two human trackways (Fig. 3a). Deformation in front of one of the mammoth’s manus tracks partially closed two tracks of the northbound human trackway, showing that the mammoth crossed that human trackway after it was made. In turn, a single human footprint of the southbound human trackway is superimposed on a mammoth manus track, showing that the human crossed the mammoth track on the return. This provides a clear sequence for the tracks, demonstrating co-association. Though the time-lapse between each of the three track-making events is unknown, the mammoth track is temporally book-ended by the two human trackways (believed to be the same individual). Current N. American dates for an initial human presence along with dates for mammoth extinction on the continent support a late Pleistocene biostratigraphical dating of the tracks at WHSA^7,14^.

## Results

**Figure 4:**
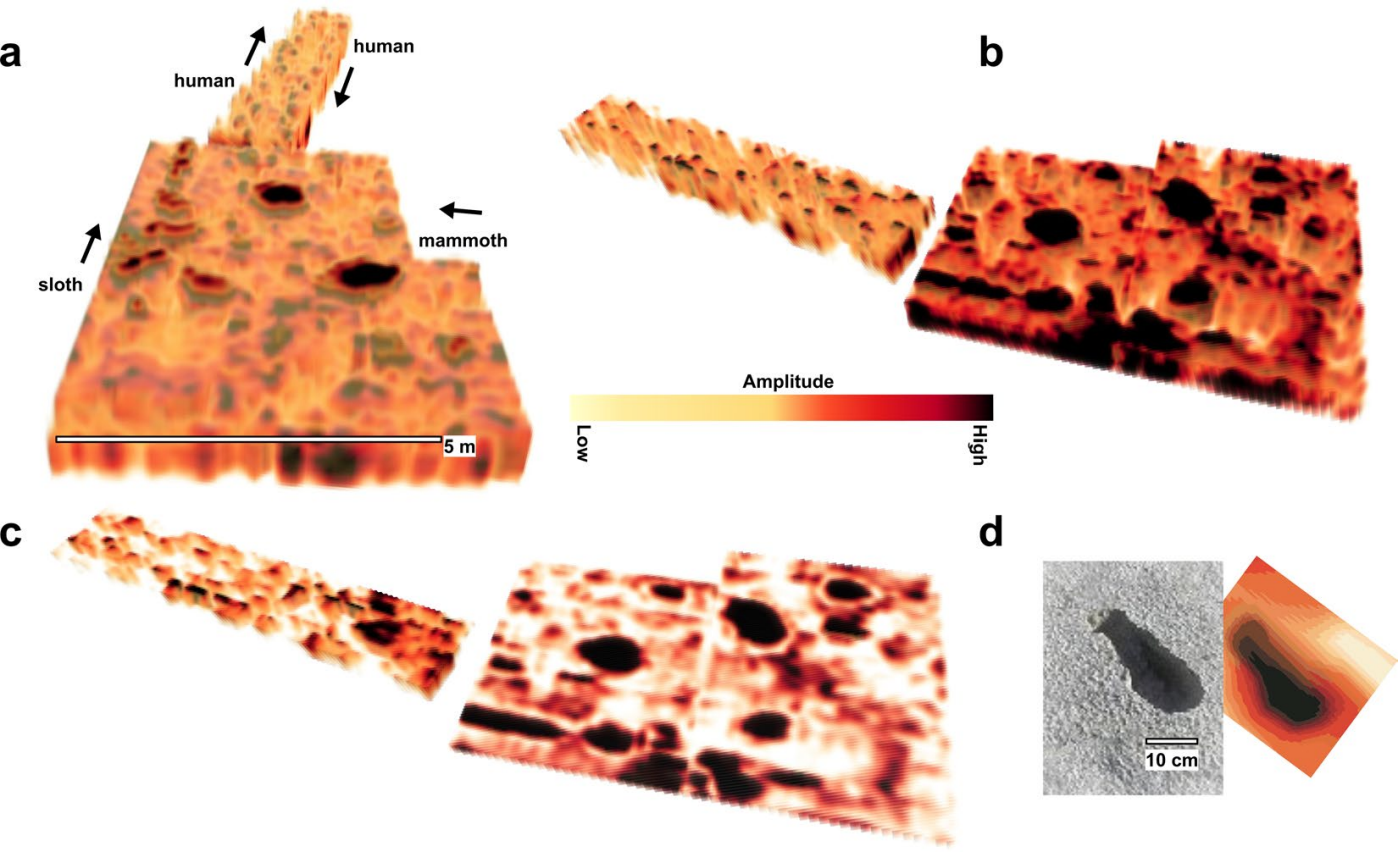
3-D perspective-view GPR results at various depths revealing hidden tracks and volumetric variations including sub-track consolidation. (**a**) Just beneath the surface (0–5 cm). (**b**) 5–10 cm of surface clipped. (**c**) 10–15 cm of surface clipped. (**d**) A photograph of an excavated human print (left) shown beside a close-up view of the corresponding GPR anomaly (right) at a depth of 5–10 cm, collected prior to excavation.

Location-1: The results of our GPR survey (Fig. 3; see Material and Methods section for parameters) demonstrate that human footprints can be identified with GPR with horizontal resolution sufficient to estimate stride-length (Fig. 4). Table 1 compares the stride and step estimates measured after excavation to those estimated from GPR data at Location-1. The results are broadly comparable and significantly show similar variance. The GPR survey only failed to identity one human track out of the 27 present at Location-1. The survey line interval of 12.5 cm meant that occasionally a human track could be entirely missed or insufficiently sampled, suggesting that 100% successful detection could be achieved thorough minor modifications to the survey design. Importantly, the survey was able to resolve targets for excavation that are not visible at the surface (Figs 3, 4). Such areas warrant further investigation in the future.

**Table 1:** Human foot size, stride and step length calculations derived via the GPR survey for Location 1.

	GPR Derived (mm)			Field Derived (mm)		
	Mean	Standard Deviation	N	Mean	Standard Deviation	N
S Bound Foot Size	281.23	2.38	8	266.78	8.31	15
N Bound Foot Size	281.8	2.13	12	257.02	8.02	13
North Step Length	804.7	1.53	12	712.52	2.89	16
South Step Length	798.02	2.62	10	693.14	2.22	14
North Stride Length	1463.33	2.43	12	1422.02	4.21	16
South Stride Length	1451	4.35	9	1404.78	4.18	14

The field derived track data are based on field survey of excavated tracks using a grid and off-set method, while the GPR derived track data are based on GPR anomalies marked for Location 1 (Fig. 3b).

Further, certain 3-D properties of the human footprints also appear to be resolvable within the GPR data, with the prints exhibiting detectable variation with depth (Fig. 4). Detailed topological information for the plantar surface of human tracks is beyond the resolution of the present data set, but for the majority of tracks visible, both the presence and relative depth could be inferred from the GPR data. Precise GPR velocity estimates would be needed to provide accurate absolute depth estimates from two-way travel times. Comparison of excavation depths of tracks with GPR data, however, suggests a velocity of 0.05–0.06 m/ns, a normal range for a moist, fine-grained substrate. The potential here for future development is clear and with higher antenna frequencies, closer survey line spacing, and transillumination (multi-directional data collection), improved 3D resolution should be achievable, including potentially topological information for the plantar surface of smaller tracks, (e.g., human ones).

**Figure 5:**
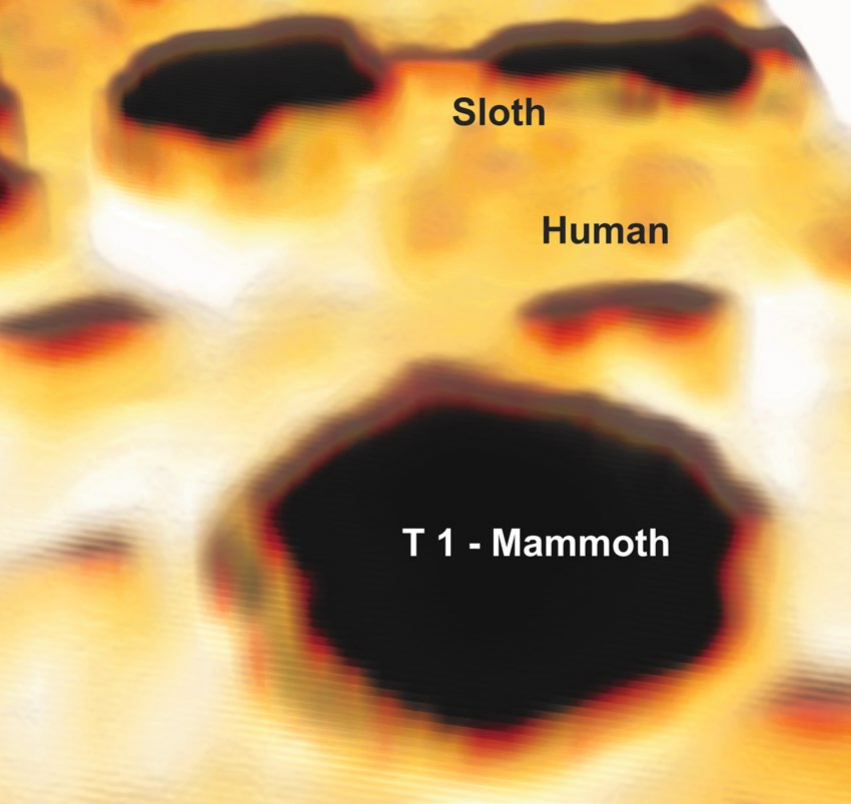
Close up 3-D GPR perspective of mammoth track 1 (T 1) along with human and sloth tracks. The presence of sloth tracks was not known prior to the GPR survey and was later confirmed when the prints became partially visible after a period of precipitation. The sloth prints remain unexcavated at this location.

**Figure 6:**
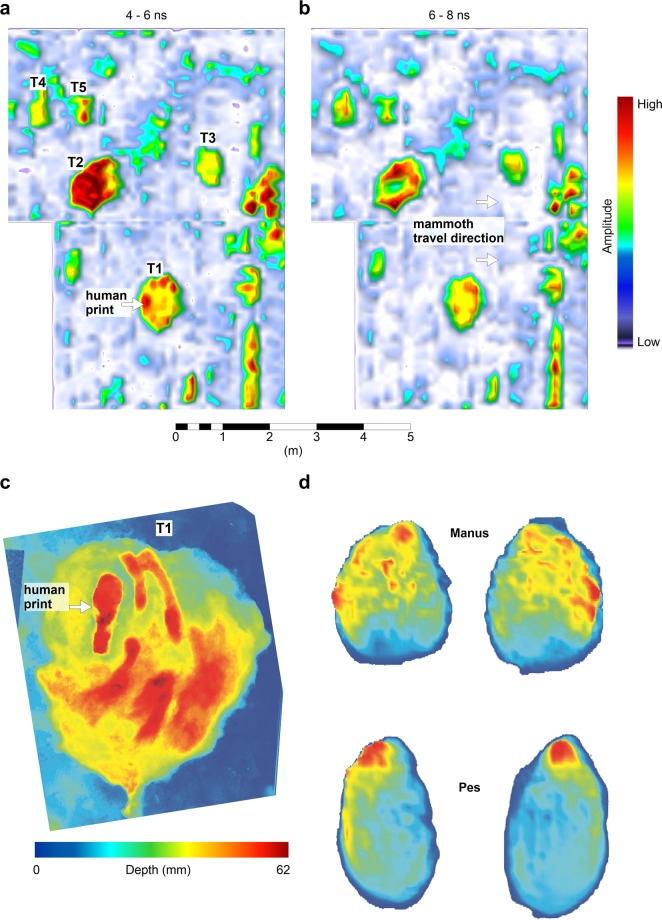
Location- 2 GPR slices at given travel-times with comparative data. Each 2 ns slice represents an estimated thickness of 5–6 cm. (**a**) Bottom or near bottom of true tracks, with a superimposed human footprint seen in the perimeter of T1. (**b**) Slice immediately beneath the tracks yield amplitude patterns similar to observed plantar pressure data. (**c**) Depths derived from excavation of the largest mammoth track (T1) with human print. (**d**) Mean plantar pressure data from African elephants (Loxodonta africana), curtesy of Panagiotopoulou *et al*.^20^. This based on the mean records of five elephants and the methods are described in Panagiotopoulou *et al*.^20^. Means for each of the five animals are based on between 1 and 24 pressure records.

Location-2: Both mammoth and human tracks have been identified and excavated at Location-2, along with a range of other potential tracks which have yet to be excavated. For example the anomalies at the western side of Location-2 (Figs 3, 4) provided future excavation targets that were not visible in any form at the time of the survey. These were later determined to be sloth tracks after becoming partially visible following a period of rain (though interpretation is preliminary as these remain unexcavated) (Fig. 5). The largest of the mammoth footprints (837 × 714 mm; Fig. 3, T1) has been excavated (Fig. 6c). Deformation in front of this track partially closed the northbound human tracks 200–300 mm to the west. The track was subsequently overprinted by one of the south bound human tracks (Fig. 6a,c). The exact outline of the mammoth track, believed to be made by a manus, is not well-defined but by analogy with extant *Loxodonta africana* would most likely correspond to a mature (>50 years) bull with a potential shoulder height of over 3 metres^15–17^.

**Figure 7:**
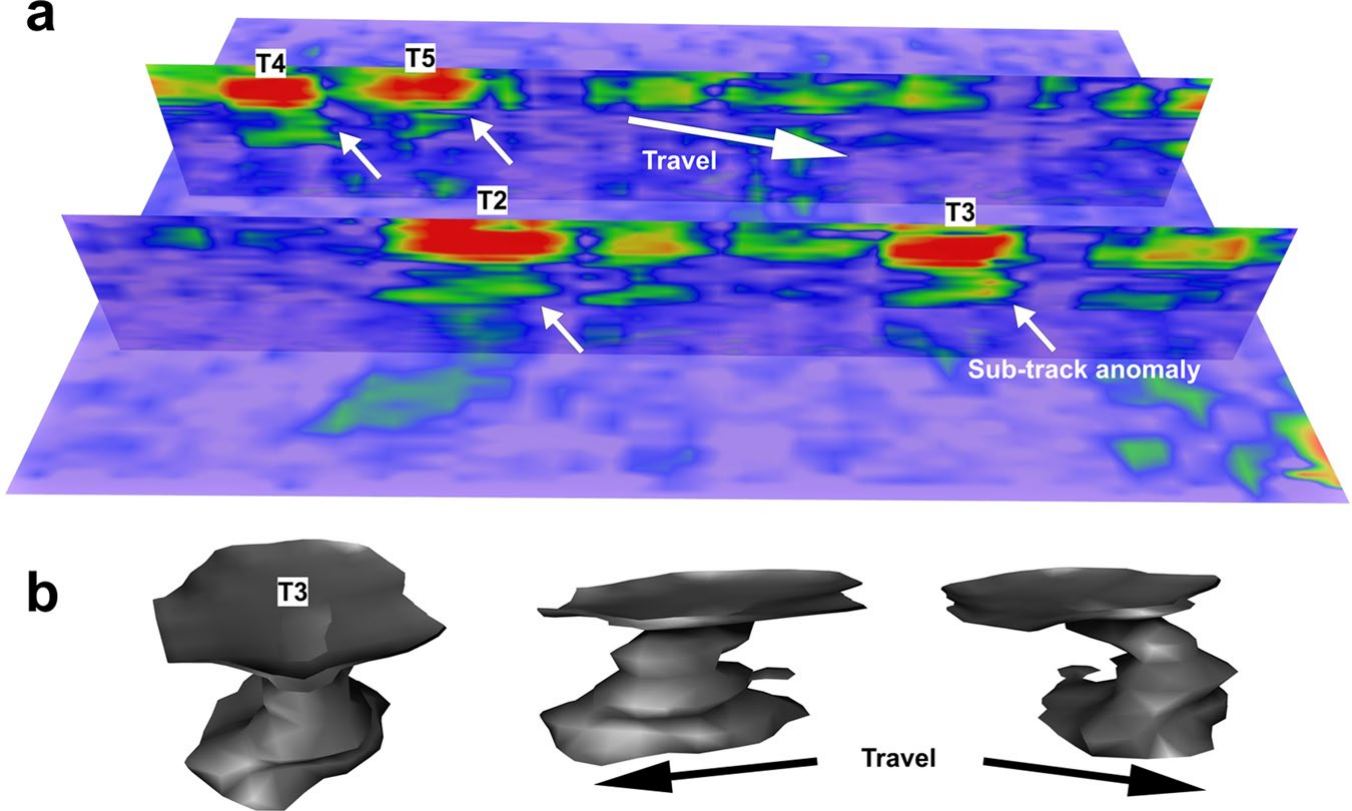
(**a**) GPR fence diagram showing amplitude variations below the mammoth tracks. Note the ‘hook-shaped’ structure indicated by the white arrows consistently points in the mammoth’s travel direction. (**b**) GPR amplitude isosurface from T3 rotated to show several perspectives of the 3-D structure of the sub-track anomaly.

Deformation structures below and around mammoth tracks elsewhere at WHSA have been investigated using a series of hand-dug trenches. Deformation occurs initially through loading below the anterior part of the track-maker’s foot, followed by a posterior-directed shear component as toe-off occurs, before finally pressure release induces fluid escape. The specific structures present vary with sub-surface stratigraphy but are always driven by anterior loading. These observations compare well to mean plantar pressure data obtained for African elephants (*Loxodonta africana*) trained to walk across force plates^18^. For African elephants mean plantar pressure peaks in the lateral and distal parts of the foot^19,20^ which corresponds to areas of maximum sub-track deformation^18^. Figure 6 shows two depth slices through location-2; the bottom or near bottom of the tracks appear in Fig. 6a (4 to 6 ns), also showing a human print in track 1 (T1), which is further illustrated in Fig. 6c. At depths just below (6 to 8 ns) shown in Fig. 6b, GPR amplitude variations are visible across the mammoth tracks, with higher amplitudes in the anterior and lateral sides of individual tracks. This layer corresponds to that immediately below the surface of the true track. This is broadly similar to the plantar pressure observed by Panagiotopoulou *et al*.^20^ for extant African elephants (*Loxodonta africana*; Fig. 6d). In vertical cross section (Fig. 7a) the amplitude pattern continuing below the tracks has a hook-shape with the apex at the anterior side of the track; that is in the direction of travel. Bennett *et al*.^18^ have shown that this corresponds to an area of peak deformation below mammoth tracks at WHSA due to the biomechanics of the locomotion. The patterns reported here are consistent across all the mammoth tracks in the surveyed area (Fig. 7a,b), with a consistent 3-D structure (Fig. 7b). We suggest that variations in amplitude with depth are detecting sediment compression consistent with the plantar pressure records for extant Proboscidea. Deformation by the weight-load of the mammoth would cause compression of the substrate and in theory increase electrical permittivity in the compacted sediment, by reducing the low-permittivity air-fraction per unit volume. In turn this should increase the surface area per unit volume, along with associated soil moisture, and the net result is a slight reduction in radar velocity resulting in higher amplitude GPR reflections where the compacted substrate occurs. We suggest therefore that the GPR data is picking up some semblance of a plantar pressure record for the extinct mammoth.

## Discussion

We have recently reported on the use of magnetic sensors to detect mammoth foot prints at WHSA^11^ and the limitations reported in that work are overcome by the use of GPR in the work reported here. First, the magnetometer is less reliable in detecting smaller tracks such as those made by humans. Human tracks are only detected if the base of the true track (plantar contact surface) is deeply impressed relative to the surface (>200 mm). Track depth and therefore fill-volume appears critical. Mammoth tracks are always detected, however. The instrument is also subject to intermittent external electromagnetic noise from nearby military activity which is specific to WHSA but none the less important. Lastly, magnetic data are not well-suited to providing 3D data, which are especially valuable in instances where tracks comprise a palimpsest of overwritten track-making events^7^. It does, however, offer a method of broader prospection that could be applied where there are surface-evident tracks, or for general reconnaissance of suspected track locations, particularly for larger fauna. For example, shore normal survey transects at playas and former lake beds should in theory identify tracks by analogy with the types of distribution described in recent field observations^21,22^.

GPR surveying, on the other hand, was able to resolve 96% of the human tracks present and all of the larger vertebrates. Results suggest, however, that with refined survey design all human tracks could be detected simply as a matter of scaling. The tracks are detectable due to the infill which exhibits higher amplitude GPR reflections than the surrounding substrate. This contrast is likely due to stronger electrical properties within the tracks than in the surrounding sediment matrix and the higher electrical permittivity probably reflects a difference in water content due to textural contrasts. In this case, the GPR response suggests that the substrate filling the prints holds more moisture than the surrounding sediment even under dry conditions, something that is evident when the tracks are excavated.

However, it is the subsurface amplitude variations below the mammoth tracks that we consider to be of particular significance. These are manifest as areas of higher amplitude that likely have a different explanation from that postulated above for the prints themselves. With the sub-track anomalies, it is not newly introduced sediments that explain the reflections, but we theorize this is caused by compression of the existing sediments which alters the electrical permittivity. The similarity between the plantar pressure records of extant elephants and the areas of anomalous high amplitude immediately beneath the area the footprint is striking, and suggests GPR is detecting a plantar pressure record below the mammoth track due, we suggest, to compression of sub-track sediment. This proposed compression pattern was not visible with excavation, and appears at least at the tested location to be only discernible with GPR.

Conventional biomechanical inference from footprints often relies on a pressure to depth substitution in which deeper areas reflect higher plantar pressures. This has been found to only hold however for shallow tracks in the case of human footprints^23^, but is something that is rarely questioned in ichnological studies for larger or extinct trackmakers. Other experimental studies that have examined this relationship in modern human footprints have also found the relationship to be tenuous^24,25^. There are many reasons why strain may not be solely accommodated by footprint volume (i.e. depth), and moreover taphonomic processes can rapidly modify observed footprint topology obscuring any relationship^26^. For example, the topology of the manus track (T 1) in Location-2 (Figs 2b, 6c) does not reflect the sub-surface pressure anomalies (Fig. 6b). Our results suggest that irrespective of variation in track depth, a pressure record is encoded via sub-track sediment properties (likely compression), and in some cases this is independent of the track and its topology.

Taken to its logical extent, potentially thousands of plantar pressure records are waiting therefore to be collected at sites like WHSA and elsewhere in North America^1,7^ and Namibia^26,27^ without the need for tracks to be excavated. The potential here to enhance our understanding of the biomechanics of extinct animals may yield important information for developing more sophisticated biomechanical models from and for extant elephants and by analogy from anatomically similar dinosaurs such as sauropods^28^. It may also improve the quality of geotechnical models applied to both elephants and mammoth tracks since it would allow estimated plantar pressures to be used rather than as now uniform indenters^29^.

At sites such as White Sands, a new data archive in the form of a rare and unique ichnological record is there to be unlocked. Accessing this record in all conditions using non-destructive geophysical methods has significant implications for the effectiveness of research, conservation and resource management at White Sands and beyond. Knowing where unexcavated tracks are located is a key step in management, monitoring and prioritization for resource conservation, especially since excavation leads ultimately to loss. With higher resolution surveys possible, we anticipate in the future that excavation may not always be necessary on an extensive scale for study of such tracks. Unpublished experimentation with different antenna frequencies has shown that the most consistent results are obtained with a 250 Hz antenna, at least at WHSA where the substrate exhibits high electrical conductivity, and that enhanced resolution can be obtained by increasing the grid resolution and combining perpendicular lines. Our unpublished tests with higher antenna frequencies (500 MHz and 1 GHz) show some promise in obtaining finer detail on smaller tracks in some cases, such as those of humans, which will be the subject of a future paper. Irrespective of this, the power of the approach lies not just in imaging buried track topology but in the *additional* information obtained on sub-track compression. So beyond the immediate and obvious benefits of locating and imaging the tracks themselves, GPR offers ancillary information on pressure and momentum due to detectable effects on sediments below and around the tracks. Resolvable and consistent trends in the GPR data suggest that each footprint has an associated sub-structure caused by compaction of surrounding sediment. Initial findings suggest that this is related both to compression from the weight of the track-maker, along with shearing forces from the momentum of the trackmaker. Therefore, information about the size and direction of the trackmaker are likely exhibited in the broader GPR patterns, offering a previously uninvestigated avenue into the biomechanics of extinct species such as the mammoth. This has implications for the study of fossil tracks well beyond White Sands, including the possibility that under suitable conditions these sub-track compaction contrasts could be retained after lithification and therefore be present below the tracks of dinosaurs or other extinct vertebrates.

## Materials and Methods

The study site was selected from the total length of the double trackway (>800 m) on the basis of having a combination of different animal tracks. The area to be surveyed was staked out and photographed before and after excavation using a monopole. Ortho-rectified photo mosaics were made using Agisoft Photoscan Pro (v. 1.5.0; www.agisoft.com). Tracks were excavated with brushes and dental picks. Once excavated 3D models of the tracks were made using DigTrace (www.digtrace.com) which uses OpenMVG an open-source structure from motion photogrammetry engine. Plantar pressure records were proved by Olga Panagiotopoulou^19^. This consisted of multiple plantar pressure records for each foot of 5 African elephants collected using a Zebris Medical plantar pressure platform. Means for each foot were co-registered and combined to produce the data.

GPR equipment included a Noggin series 250 MHz unit by Sensors and Software Inc. (https://www.sensoft.ca/products/noggin/overview/*)*. Instrument parameters included a 120 nanosecond time-window, 8 stacks, and 5 cm trace interval. GPR data processing was done with EKKO_View; EKKO_mapper (Sensors and Software Inc.) and included dewow, gain, and envelope. GPR images were produced with VOXLER 4 and Surfer 14 by Golden Software Inc. www.goldensoftware.com/products

10 mm thick interlocking foam mats were used to cover the site in sections for the geophysical survey. These were pre-marked with lines at 12.5 cm intervals. The GPR line spacing of 12.5 cm gave coverage of 18 m²/hr, for parallel transects, inclusive of setting up and moving the protective foam pads. Although intensive, this provided a fairly rapid and non-destructive option to gather information where tracks are suspected. Once excavated, these tracks begin to degrade immediately and need to be digitized in 3D (via photogrammetry, or optical laser scanner), or physically cast for conservation purposes. By comparison, excavation and 3D capture, via photogrammetry, of Location-1 took approximately 26 hours or 0.15 m²/hr.

## Data Availability

Data related to this paper may be requested from the authors.
